# Impact assessment of [ ^18^ F]FDG PET/CT in predicting EGFR gene mutation status in patients with lung adenocarcinoma using recovery coefficient-based correction of semi-quantitative and volume-based PET metrics

**DOI:** 10.1055/a-2777-2278

**Published:** 2026-04-13

**Authors:** Aleksei Leontev, Angelina Lokhova, Aleksandr Khalimon, Malika Khodzhibekova, Tatyana Lazutina, Gulnara Khamadeeva, Daria Khodakova, Irina Pylova, Vitaly Bobrov, Leila Atakishieva, Natalia Kadymova, Anastasia Nikiforuk, Bogdan Shvets, Nadezhda Volchenko, Viktoriya Surkova, Petr Shatalov, Andrey Kaprin

**Affiliations:** 1 Department of Nuclear MedicineP. Hertsen Moscow Oncology Research Institute – the branch of the FSBI “National Medical Research Radiological Centre” of the Ministry of Health of the Russian FederationMoscowRussian Federation; 2 Department of Nuclear MedicineN. Lopatkin Scientific Research Institute of Urology and Interventional Radiology – the branch of the FSBI “National Medical Research Radiological Centre” of the Ministry of Health of the Russian FederationMoscowRussian Federation; 3 National Medical Research Radiological CentreObninskRussian Federation; 4 Medical Institute of Peoples’ Friendship University of RussiaMoscowRussian Federation

**Keywords:** lung adenocarcinoma, epidermal growth factor receptor, PET/CT, [ ^18^ F]FDG, recovery coefficient, SUV harmonization

## Abstract

**Aim:**

The aim of this study was to assess the impact of [ ^18^ F]FDG PET/CT in predicting EGFR gene mutation status in patients with lung adenocarcinoma using recovery coefficient-based (RC-based) correction of semi-quantitative and volume-based PET metrics.

**Methods:**

A retrospective multi-center study included 63 patients diagnosed with lung adenocarcinoma who underwent [ ^18^ F]FDG PET/CT. Acquisition was performed according to the EANM guidelines using two EARL-accredited PET/CT-systems. All patients were divided into an EGFR-mutant (n=30) and a wild-type (n=33) groups based on the results of molecular testing. Maximal diameter and CT volume of each primary tumor lesion were measured. [ ^18^ F]FDG uptake was evaluated by measurement of semi-quantitative and volume-based PET metrics such as SUVmax, SUVpeak, MTV, and TLG. TLG was measured using PET segmentation method by 3D-Isocontour tool with a 41% of SUVmax threshold (TLG _41%_ ) and a threshold adapted to the CT volume (TLG _CT_ ). Additionally, TLR was calculated as the ratio of tumor SUVpeak to liver SUVmean. Acquisition data of the NEMA IEC Body Phantom Set NU2–2018 were applied for plotting the RC curves which were used for following RC-based correction of PET metrics.

**Results:**

No significant difference in tumor lesion maximal diameter, CT volume, evaluated values of PET metrics before and after RC-based correction was found between the EGFR-mutant and wild-type groups (p > 0.05).

**Conclusion:**

Observed semi-quantitative and volume-based PET metrics obtained from [ ^18^ F]FDG PET/CT have no significant value for predicting EGFR gene mutation status in patients with lung adenocarcinoma, regardless of whether RC-based correction is applied.

## Introduction

Lung cancer is a widely prevalent malignant neoplasm and the leading cause of cancer-related mortality among adults worldwide. In 2022, there were 2.5 million new cases of lung cancer and 1.8 million deaths attributed to this disease, representing 12.4% of all malignant neoplasms and 18.7% of all cancer-related deaths, respectively 1 . In Russia, over the past decade, lung cancer has been consistently ranked as the leading cause of cancer-related mortality among adults, accounting for 17.6% of all cancer-related deaths in 2023 2 .

Non-small cell lung cancer (NSCLC) is the most common type of lung cancer (80–90%). The main histological subtypes of NSCLC are adenocarcinoma (40%), squamous cell carcinoma (25–30%), and large cell carcinoma (10–15%). When adenocarcinoma is identified, molecular testing of tumor or blood samples is recommended to identify gene mutations, particularly in the epidermal growth factor receptor (EGFR) gene 3 .

EGFR, also known as ErbB1/HER1, is a transmembrane tyrosine kinase physiologically expressed on the epithelial cell membranes. Ligands such as epidermal growth factor, transforming growth factor alpha, and amphiregulin induce the homo- or heterodimerization of EGFR, followed by autophosphorylation of the C-terminal tail of its intracellular domain 4 . This leads to the activation of signaling pathways, primarily RAS-RAF-MEK-ERK/MAPK, PI3K/AKT/mTOR, and JAK/STAT, that are involved in mediating the cellular processes induced by the mitogenic signals of EGFR ligands.

NSCLC cells are characterized by constitutive EGFR activity caused by mutations in exons 18–21 of the gene encoding the receptor's tyrosine kinase domain. It leads to the induction of cellular invasive growth, proliferation, tumor angiogenesis, and inhibition of cellular apoptosis. Hyperactivation of these processes can also be caused by EGFR overexpression on the NSCLC cell membranes, identified in 89% of squamous cell carcinoma and in 41% of adenocarcinoma 5 . The discovery of activating mutations in the EGFR gene has significantly changed the NSCLC treatment approach by introducing targeted therapy with tyrosine kinase inhibitors (TKIs) into clinical practice, leading to increased rates of objective response, progression-free survival, and overall survival.

The exon 19 deletion (Del19) and the p.L858R point mutation in exon 21 are the most prevalent mutations in the EGFR gene associated with the sensitivity to TKIs, accounting for 45% and 40–45% of observed mutations in EGFR gene, respectively. Other mutations associated with the sensitivity to TKIs, such as the p.G719X, p.L861Q, and p.S768I point mutations, as well as exon 19 insertions, are less common (1–5%). The exon 20 insertions (except for the A763_Y764insFQEA) and the p.T790M point mutation are associated with primary and acquired resistance to TKIs. The last-mentioned point mutation is the most common cause of acquired resistance to first- and second-generation TKIs, that has been overcome with development of third-generation TKIs (osimertinib) and their approval for clinical use 6 . The above-mentioned genomic rearrangements are more frequently observed in female never-smoker Asian patients 7 .

Molecular testing of histological samples obtained from the primary tumor or metastatic lesions by surgery or biopsy is traditionally performed to determine EGFR gene mutation status. Cytological or blood samples may be the acceptable alternatives in the absence of histological samples. However, molecular testing of blood samples is characterized by low specificity and high cost. In case of centrally located tumors, biopsy is performed as part of bronchoscopy. Transthoracic or, if this approach is ineffective, thoracoscopic biopsy is performed for peripheral lesions that are typical for adenocarcinoma subtype. Despite the minimal invasiveness of transthoracic and thoracoscopic biopsies, these procedures are associated with complications, for instance hemorrhage and wound infection. In some cases, such as uninformative tumor samples or disease progression caused by acquired resistance to first- and second-generation TKIs, a rebiopsy is necessary. In this regard, molecular imaging could serve as a viable alternative to the established methods for determining the EGFR gene mutation status.

Currently, X-ray is the routinely performed modality for chest imaging that could be supplemented by computed tomography when lung cancer is suspected. 2-[ ^18^ F]fluoro-2-deoxy-D-glucose ([ ^18^ F]FDG) positron emission tomography combined with computed tomography (PET/CT) shows high diagnostic accuracy in primary staging, restaging after neoadjuvant chemotherapy or chemoradiotherapy, relapse detection, and evaluation of the treatment response in patients with NSCLC. However, [ ^18^ F]FDG PET/CT demonstrates lower accuracy than magnetic resonance imaging in detecting brain metastases 8 .

Exploring the predictive value of [ ^18^ F]FDG PET/CT in oncological patients is one of the areas of interest in nuclear medicine due to its non-invasiveness and a wide range of analyzed parameters. Over the past decade, there has been a stable interest in the scientific community regarding the role of [ ^18^ F]FDG PET/CT for predicting EGFR gene mutation status in patients with newly diagnosed NSCLC 9 10 11 12 13 14 15 16 17 18 19 20 21 22 . However, these studies had several limitations, including the impact of the partial volume effect (PVE) on the quantitative assessment of [ ^18^ F]FDG uptake in small tumor lesions, as well as SUV overestimation, for example, when using point spread function (PSF) modeling. It may explain the lack of consensus and unambiguous results of these studies, which served as the basis for present research, supplemented by recovery coefficient-based correction (RC-based correction).

## Materials and methods

The Ethics Committee of the P. Hertsen Moscow Oncology Research Institute and N. Lopatkin Scientific Research Institute of Urology and Interventional Radiology – the branches of the FSBI “National Medical Research Radiological Centre” of the Ministry of Health of the Russian Federation has confirmed that no ethical approval is required. All patients provided written informed consent prior to enrollment.

This retrospective bicenter study included 63 patients (33 males and 30 females) diagnosed with lung adenocarcinoma who underwent [ ^18^ F]FDG PET/CT from January 2023 to February 2025 at P. Hertsen Moscow Oncology Research Institute and N. Lopatkin Scientific Research Institute of Urology and Interventional Radiology – the branches of the FSBI “National Medical Research Radiological Centre” of the Ministry of Health of the Russian Federation (36 and 27 patients, respectively) ( Table 1 ). The mean age of the patients was 66±10.5 years. I, II, III, and IV AJCC (American Joint Committee on Cancer) stages were observed in 16 (25.4%), 10 (15.9%), 14 (22.2%), and 23 (36.5%) patients, respectively. The inclusion criteria were histologically confirmed lung adenocarcinoma with subsequent molecular testing for EGFR gene mutation status, the absence of previous NSCLC treatment, satisfactory fusion of PET and CT images, the ability to perform automatic segmentation of primary tumor lesion on either PET or CT images, or to measure its mutually perpendicular diameters. Tumor lesion cavitation, determined by CT features, was the exclusion criterion. All patients were divided into an EGFR-mutant (n=30) and a wild-type (n=33) groups based on the results of molecular testing of the histological samples obtained from the primary tumor or metastatic lesions by surgery or biopsy.

**Table:** **Table 1** Patient characteristics.

Characteristic	Patient group	
	EGFR-mutant, n=30	Wild-type, n=33
Age, years	68.9±7.5	61.2±11.7
Gender:		
male female	7 (23.3%) 23 (76.7%)	25 (75.8%) 8 (24.2%)
Smoking history:		
never-smoker ever-smoker	22 (73.3%) 8 (26.7%)	9 (27.3%) 24 (72.7%)
AJCC stage:		
I II III IV	8 (26.7%) 5 (16.7%) 6 (20%) 11 (36.6%)	8 (24.2%) 5 (15.2%) 8 (24.2%) 12 (36.4%)
Grade:		
G1 G2 G3 not determined	2 (6.7%) 12 (40%) 12 (40%) 4 (13.3%)	0 12 (36.4%) 12 (36.4%) 9 (27.2%)
EGFR gene mutation:		
Del19 p.L858R p.L861Q Ins20	14 (46.6%) 12 (40%) 2 (6.7%) 2 (6.7%)	Not applicable

[ ^18^ F]FDG was administered intravenously at a dose of 220 MBq per 1 m ^2^ of the patient’s body surface area by the MEDRAD Intego PET Infusion System (Bayer, Germany). The mean administered activity was 407.3±47.1 MBq. Acquisition was performed in accordance with the European Association of Nuclear Medicine (EANM) guidelines using two EARL-accredited (EANM Research Ltd) 5-ring BGO PET/CT-systems Discovery IQ Gen 2 (GE HealthCare, USA). PET was performed from vertex to the feet, with 3 minutes per bed position at the trunk, 2 minutes – at the head, neck and hips, 1.5 minutes – at the upper extremities, lower legs and feet. Correction of PET data was performed using non-contrast CT. The BSREM (block sequential regularized expectation maximization) algorithm with the β-value 400 and 256×256 matrix was used for PET image reconstruction; these parameters were identical for both utilized PET/CT-systems and compliant with the EARL ^18^ F standard 2. CT was performed using both free-breathing method for PET and CT images fusion and deep-inspiration breath-holding method. The slice thickness and increment were 1.25 mm for free-breathing CT images, 1.25 mm and 1.0 mm, respectively, for deep-inspiration breath-holding CT images.

A workstation Syngo.via version 10.1.1.207 (Siemens Healthineers, Germany) with the MM Oncology application was utilized for image analysis. Maximal diameter (Dmax) and CT volume of each primary tumor lesion were measured using Solid Lung Lesion Segmentation tool at breath-holding CT images. [ ^18^ F]FDG uptake was evaluated by measurement of semi-quantitative and volume-based PET metrics such as SUVmax, SUVpeak, Metabolic Tumor Volume (MTV), and Total Lesion Glycolysis (TLG). SUV was normalized by lean body mass and calculated using the Janmahasatian formula. SUVmax, SUVpeak, MTV (MTV _41%_ ), and TLG (TLG _41%_ ) were measured using the 3D-Isocontour tool with a 41% of SUVmax threshold. TLG was measured using the 3D-Isocontour tool with a threshold adapted to the CT volume (TLG _CT_ ), obtained by Solid Lung Lesion Segmentation tool from a deep-inspiration breath-holding CT images. Additionally, TLR (Tumor-to-Liver Ratio) was calculated as the ratio of tumor SUVpeak to liver SUVmean measured by spherical volume of interest with 3 cm diameter. Nine EGFR-mutant patients were excluded from MTV and TLG analysis due to the inability to use automatic segmentation that resulted from the primary tumor spread to the mediastinum or lung atelectasis.

**Fig. 1:**
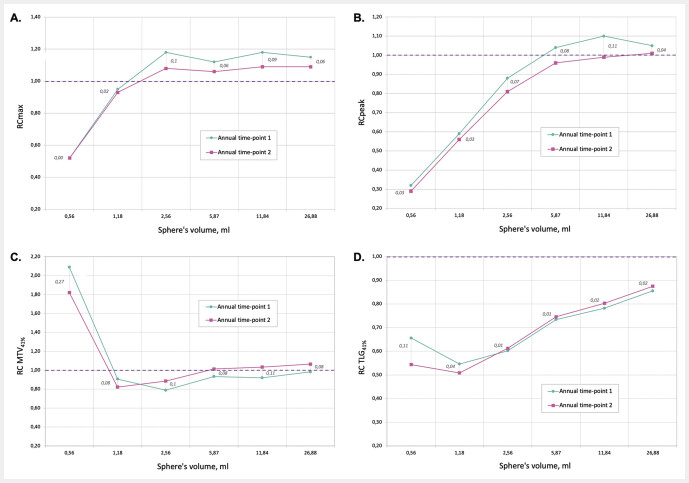
RC curves for each PET metric. SUVmax ( **a** ), SUVpeak ( **b** ), MTV _41%_ ( **c** ), and TLG _41%_ ( **d** ) RC curves derived from NEMA IEC Body Phantom Set NU2–2018 acquisition data performed at first and second annual time-points. Interannual ∆RC were calculated for each phantom’s sphere.



Due to the well-known impact of the reconstruction algorithm and its parameters on the accuracy of PET metrics it was decided to additionally correct the obtained values by considering the RC for the reconstruction used. Acquisition data of the NEMA IEC Body Phantom Set NU2–2018 obtained in accordance with EARL manual were applied for plotting the RC curves for SUVmax, SUVpeak, MTV _41%_ , TLG _41%_ , and TLG _CT_ ( Fig. 1 ). RC was calculated as the ratio of measured PET metric value to its true value. MTV _41%_ and TLG _41%_ for each phantom’s sphere were measured using 3D-Isocontour tool with 41% of SUVmax threshold. TLG _CT_ for each phantom’s sphere was measured using 3D-Isocontour tool with individual manually fitted thresholds based on the CT volume of each tumor lesion. RC-based correction was performed by dividing the measured SUVmax, SUVpeak, MTV _41%_ , TLG _41%_ , and TLG _CT_ values by RC values obtained for each tumor volume through its extrapolation to RC curves. TLR was likewise calculated using tumor SUVpeak after RC-based correction and liver SUVmean. In the analysis after RC-based correction, 9 (1 mutant and 8 wild-type) and 14 (6 mutant and 8 wild-type) patients were excluded from calculating volume-based and semi-quantitative PET metrics, respectively, due to the inability to obtain RС values. This resulted from tumor volume (range 32.4–92.7 ml) exceeding the volume of the largest phantom’s sphere, equal to 26.88 ml. After RC-based correction, bias (%) of the PET metrics was calculated for each tumor lesion across all patients, EGFR-mutant and wild-type groups using the following formula:

where *RC _non-corr_ PET metric’s value* – PET metric’s value before RC-based correction, *RC _corr_ PET metric’s value* – PET metric’s value after RC-based correction.

Additionally, the correlation analysis between the PET metrics’ bias and CT volume of the tumor lesion was performed.

Statistical analysis was performed using the SPSS Statistics 28 software package to evaluate the significance of the difference in SUVmax, SUVpeak, MTV _41%_ , TLG _41%_ , TLG _CT_ , and TLR (both before and after RC-based correction), Dmax, and CT volume of primary tumor lesion as well as in bias of the PET metrics between the EGFR-mutant and wild-type groups. All variables were first evaluated for normality using the Shapiro-Wilk test. The results were presented as median and interquartile range for PET metrics, Dmax, and CT volume, and as mean with 95% confidence interval (95% CI) for the PET metric’s bias. Comparisons of two independent groups were performed by Student’s t-test when the variables were within a normal distribution and by Mann-Whitney U-test when they were not normally distributed. Correlation analysis was performed using non-parametric Spearman correlation test to assess the relationship between the PET metric’s bias and CT volume of the tumor lesion. А *p* -value < 0.05 was defined as statistically significant.

## Results

The results are presented in Table 2 and Table 3 .

**Table:** **Table 2** Comparison of PET metrics’ values before RC-based correction, Dmax, and CT volumes of tumor lesions between two groups of patients, Me [Q0.25; Q0.75].

Patient group	SUVmax ^а^	SUVpeak ^а^	MTV _41%_^b^	TLG _41%_^b^	TLG _CT_^b^	TLR ^b^	Dmax ^а^ , cm	CT volume ^b^ , ml	*p* -value
**EGFR-mutant**	*n=30* 6.6 [3.6; 9.3]	*n=30* 5.7 [2.8; 7.6]	*n=21* 5.4 [3.3; 10.4]	*n=21* 21.5 [9.2; 37.8]	*n=21* 27.8 [11.5; 52.1]	*n=30* 3 [2; 5.2]	*n=25* 3 [2.5; 4]	*n=21* 7 [5.2; 14.7]	>0.05
**Wild-type**	*n=33* 8.5 [6.4; 10]	*n=33* 6.6 [5.2; 8.8]	*n=33* 9 [3.6; 16.2]	*n=33* 36.9 [10.2; 78]	*n=33* 54.7 [14.4; 123]	*n=33* 4 [3; 5.3]	*n=33* 3.6 [2.5; 4.4]	*n=33* 12.1 [4.6; 24.4]	
* a – Student’s t-test b – Mann-Whitney U-test *									

**Table:** **Table 3** Comparison of PET metrics’ values after RC-based correction, Dmax, and CT volumes of tumor lesions between two groups of patients, Me [Q0.25; Q0.75].

Patient group	SUVmax ^b^	SUVpeak ^b^	MTV _41%_^b^	TLG _41%_^b^	TLG _CT_^b^	TLR ^а^	Dmax ^а^ , cm	CT volume ^b^ , ml	*p-* value
**EGFR-mutant**	*n=24* 5.7 [3.2; 7.6]	*n=24* 5.2 [3; 6.8]	*n=20* 5.2 [3.3; 10.1]	*n=20* 28.9 [16; 46.4]	*n=20* 31 [14.7; 51.7]	*n=24* 3 [2.1; 4.5]	*n=25* 3 [2.5; 4]	*n=21* 7 [5.2; 14.7]	>0.05
**Wild-type**	*n=25* 6.8 [5.4; 8.1]	*n=25* 6.7 [4.7; 7.7]	*n=25* 6.2 [3.6; 11.7]	*n=25* 24.9 [12.9; 66.4]	*n=25* 35.8 [16.2; 82.6]	*n=25* 3.6 [2.7; 5]	*n=33* 3.6 [2.5; 4.4]	*n=33* 12.1 [4.6; 24.4]	
*a – Student’s t-test* *b – Mann-Whitney U-test*									

No significant difference in tumor lesion Dmax, CT volume, and obtained values of PET metrics before and after RC-based correction was found between the EGFR-mutant and wild-type groups ( *p* > 0.05).

**Fig. 2:**
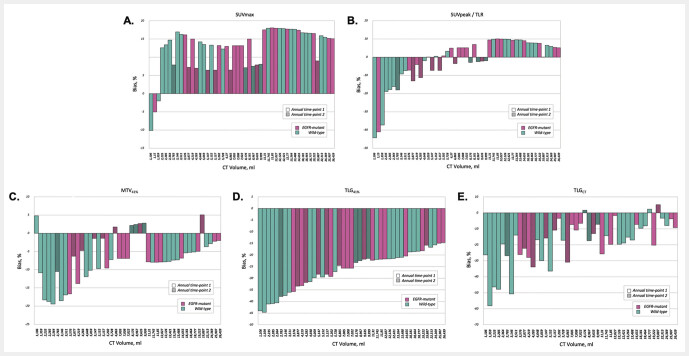
Waterfall plots of PET metrics’ bias for each tumor lesion. SUVmax ( **a** ), SUVpeak/TLR ( **b** ), MTV _41%_ ( **c** ), TLG _41%_ ( **d** ), and TLG _CT_ ( **e** ).

After RC-based correction, the PET metrics’ bias was observed across all lesions ( Fig. 2 ). Mean bias values with 95% CI were as follows: 13.1 [11.8–14.4] % for SUVmax; 9.3 [6.6–12] % for SUVpeak/TLR; 7.1 [5.6–8.6] % for MTV _41%_ ; 24.4 [21.3–27.5] % for TLG _41%_ ; 17 13 14 15 16 17 18 19 20 21 % for TLG _CT_ . Mean bias values with 95% CI in the EGFR-mutant and wild-type groups were, respectively: 12.6 [10.7–14.5] % and 13.6 [11.8–15.4] % for SUVmax; 8 [4.7–11.3] % and 10.7 [6.3–15.1] % for SUVpeak/TLR; 6.2 [4.3–8.1] % and 9 [6.7–11.3] % for MTV _41%_ ; 24.7 [21.8–27.6] % and 28.1 [24.2–32] % for TLG _41%_ ; 15.1 [10.4–19.8] % and 21.3 [14.8–27.8] % for TLG _CT_ . No significant difference in the obtained bias values was found between the EGFR-mutant and wild-type groups ( *p* > 0.05), except for the MTV _41%_ ( *p* = 0,04).

**Fig. 3:**
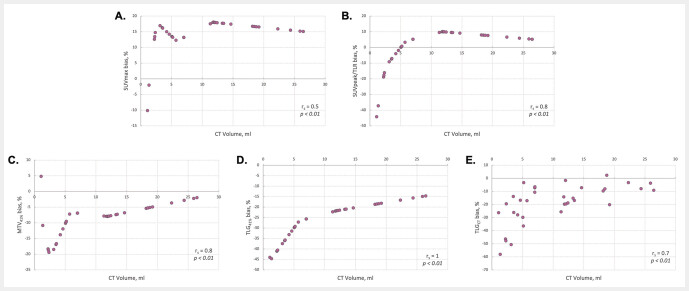
Scatter plots of tumor lesion’s CT volume and PET metric’s bias correlation. SUVmax ( **a** ), SUVpeak/TLR ( **b** ), MTV _41%_ ( **c** ), TLG _41%_ ( **d** ), TLG _CT_ ( **e** ). Only patients from the first annual time-point were included in the correlation analysis because the RCs obtained from different time-points phantom acquisition data differed.

In addition, a significant relationship between the bias and CT volume was observed ( *p* < 0.01), with: moderate positive correlation – for SUVmax (r _s_ = 0.5) and TLG _CT_ (r _s_ = 0.7); strong positive correlation – for SUVpeak/TLR and MTV _41%_ (r _s_ = 0.8) as well as for TLG _41%_ (r _s_ = 1) ( Fig. 3 ).

## Discussion

Currently, a wide range of studies dedicated to exploring the [ ^18^ F]FDG PET/CT possibilities in predicting EGFR gene mutation status in patients with newly diagnosed NSCLC has been conducted. The main objective of these studies was to determine the prognostic threshold values of PET metrics for tumor lesions, particularly SUVmax 9 10 11 12 13 14 15 16 17 18 19 20 21 , as well as MTV 22 . Conflicting results across studies indicate a lack of consensus in this area. Several authors concluded that tumor SUVmax exceeding the calculated threshold is associated with the EGFR-positive status 9 10 11 , while other studies demonstrated contrary results 12 13 14 15 16 17 18 . At the same time, authors of several studies and meta-analyses claim that there is no relationship between tumor SUVmax and EGFR gene mutation status 19 20 21 .

A retrospective study performed by Wang et al. involved 297 patients (166 males and 131 females) diagnosed with lung adenocarcinoma. Acquisition was performed using Discovery STE PET/CT-system (GE HealthCare, USA). According to the results, EGFR-mutant patients exhibited significantly lower SUVmax and smaller lesion diameter compared to the wild-type group. Mean SUVmax were 8.24±4.51 and 10.64±5.77 ( *p* < 0.001) and mean diameter were 2.89±1.46 cm and 3.54±1.9 cm ( *p* = 0.001) for the EGFR-mutant and wild-type groups, respectively. The authors concluded that the EGFR-positive status is associated with the tumor SUVmax values below the threshold of 8.2 16 .

In contrast, Ko et al., in their study, which included 132 patients (57 males and 75 females), reported that tumor SUVmax and lesion diameter greater than 6 ( *p* =0.002) and 3 cm ( *p* =0.023), respectively, were associated with the positive status of EGFR gene mutation. Acquisition was performed by Biograph family PET/CT-system (Siemens Healthineers, Germany), however the exact model of the system was not specified 10 .

In our study, not only SUVmax but also other PET metrics, such as SUVpeak, MTV, TLG, and TLR with additional RC-based correction were analyzed. SUVmax and SUVpeak/TLR measurements were overestimated in 93.9% and 57.1% of all lesions, respectively. MTV _41%_ , TLG _CT_ , and TLG _41%_ measurements were underestimated in 93.3%, 84.4%, and 100% of all lesions, respectively. RC-based correction markedly decreased the influence of technical factors (such as PVE and SUV overestimation, for example, when using PSF modeling). This allowed to obtain “true” [ ^18^ F]FDG uptake in lesions, which clearly reflects tumor biological behavior. No significant difference in PET metrics’ values both before and after RC-based correction between the EGFR-mutant and wild-type groups was found ( *p* > 0.05). This can be explained by the absence of significant difference in the PET metrics’ bias, except for the MTV _41%_ , between the EGFR-mutant and wild-type groups ( *p* > 0.05). Obtained difference in the MTV _41%_ bias ( *p* = 0.04) is close to the significance threshold and may be random because of the small patient sample size. In addition, the observed significant moderate and strong correlation between the PET metrics’ bias and CT volume of tumor lesion may explain the lack of significant difference in bias between the EGFR-mutant and wild-type groups, as no significant difference in the CT volume was found. However, our study had several limitations, including the small patient sample size which could affect the p-value; its retrospective design, which made analysis of other mutations unfeasible; and the aspherical shape of some evaluated lesions, that may lead to misapplication of RC-based correction.

Wang et al. and Ko et al. assessed only SUVmax with no RC-based correction and found a significant difference between the EGFR-mutant and wild-type groups in the comparative analysis of tumor lesion diameter. This could have been caused by SUVmax being overestimated in larger tumor lesions and underestimated in smaller ones.

Even when imaging and quality control procedures are strictly followed, SUV remains relatively variable and is also influenced by several other factors, particularly those related to the scan system such as vendor and model, acquisition specifications, as well as PET data reconstruction algorithms and parameters. Several PET scanner validation programs developed in Europe, Australia, and the United States, such as EARL (EANM Research Ltd), aim to minimize interscanner SUV variability. The authors of above-mentioned studies reported different prognostic SUVmax thresholds (i.e. 8.2 and 6). This means that the values may be obtained incorrectly due to the lack of scan systems harmonization.

RC-based correction and quantitative PET harmonization are two normalization methods aimed at reducing interscanner SUV variability, as well as increasing the reliability and reproducibility of quantitative assessment in multi-center studies. A key advantage of the first method is that it obtains values close to the true ones, thereby eliminating the dependence of SUV on the lesion size. However, its application involves a constant presence of measurement bias, primarily caused by the aspherical shape of lesions, as well as additional labor time costs for calculating the RC for each lesion. In addition, the RCs obtained from phantom acquisition data at different time-points varies, which also affects the measurement bias. Due to its lower labor intensity, an attractive alternative to the first method is SUV harmonization. At the same time, this method is also associated with a systematic measurement bias that remains stable across harmonized scan systems but does not allow for reliable quantitative assessment of the pathophysiological process. Thus, SUV harmonization, unlike RC-based correction, cannot reduce the dependence of SUV on the lesion size.

Unlike Wang et al. and Ko et al., Caicedo et al. analyzed not only EGFR gene but also KRAS (Kirsten rat sarcoma viral oncogene homologue) gene mutation status. A total of 102 patients were divided into three groups: KRAS-mutant regardless of EGFR gene mutation status (KRAS+), EGFR-mutant regardless of KRAS gene mutation status (EGFR+), and wild-type, comprising patients without rearrangements in either gene. EGFR+ patients exhibited significantly lower tumor SUVmax, SUVpeak, and SUVmean compared to EGFR– patients, comprising KRAS+ and wild-type patients ( *p* < 0.05). Comparison of tumor [ ^18^ F]FDG uptake between EGFR+ and KRAS+ patients revealed similar results ( *p* < 0.001). However, no significant difference was found between EGFR+ and wild-type patients in the comparative analysis of PET metric values of tumor lesions ( *p* > 0.05). Thus, the authors concluded that the statistical significance of the initial values of the PET metrics comparison between EGFR+ and EGFR– patients was affected by the inclusion of KRAS+ patients in the EGFR– group 20 .

## Conclusion

Our results suggest that the observed semi-quantitative and volume-based PET metrics obtained from [ ^18^ F]FDG PET/CT have no significant value for predicting EGFR gene mutation status in patients with newly diagnosed lung adenocarcinoma, even when RC-based correction is applied. Considering the limitations of our study, further multi-center studies involving RC-based correction of PET metrics, along with a comprehensive analysis of all NSCLC-associated genetic rearrangements, are required.

**Ethics approval:** The Ethics Committee of the P. Hertsen Moscow Oncology Research Institute and N. Lopatkin Scientific Research Institute of Urology and Interventional Radiology – the branches of the FSBI “National Medical Research Radiological Centre” of the Ministry of Health of the Russian Federation has confirmed that no ethical approval is required.

**Data Availability:** The datasets used and/or analyzed during the current study are available from the corresponding author on reasonable request.
